# Detecting Pre-Analytically Delayed Blood Samples for Laboratory Diagnostics Using Raman Spectroscopy

**DOI:** 10.3390/ijms24097853

**Published:** 2023-04-25

**Authors:** Pascal Hunold, Markus Fischer, Carsten Olthoff, Peter W. Hildebrand, Thorsten Kaiser, René Staritzbichler

**Affiliations:** 1 Institute of Laboratory Medicine, Clinical Chemistry and Molecular Diagnostics, University Hospital Leipzig, 04103 Leipzig, Germany; 2 Institute for Medical Physics and Biophysics, Leipzig University, 04107 Leipzig, Germany; 3 University Institute for Laboratory Medicine, Microbiology and Clinical Pathobiochemistry, University Hospital OWL of Bielefeld University, Campus Klinikum Lippe, 32756 Detmold, Germany

**Keywords:** laboratory medicine diagnostics, preclinical delays, sample age, quality assurance, Raman spectroscopy

## Abstract

In this proof-of-principle study, we systematically studied the potential of Raman spectroscopy for detecting pre-analytical delays in blood serum samples. Spectra from 330 samples from a liver cirrhosis cohort were acquired over the course of eight days, stored one day at room temperature, and stored subsequently at 4 °C. The spectra were then used to train Convolutional Neural Networks (CNN) to predict the delay to sample examination. We achieved 90% accuracy for binary classification of the serum samples in the groups “without delay” versus “delayed”. Spectra recorded on the first day could be distinguished clearly from all subsequent measurements. Distinguishing between spectra taken in the range from the second to the last day seems to be possible as well, but currently, with an accuracy of approximately 70% only. Importantly, filtering out the fluorescent background significantly reduces the precision of detection.

## 1. Introduction

Delays prior to laboratory examination are of particular importance in many fields of medicine, impacting the validity of the patient’s laboratory diagnostics, and have to be avoided to ensure timely therapy. Medical laboratories are the basis for the In Vitro Diagnostic Device Regulation of the European Union. The guideline emphasizes the obligation of the laboratory to ensure the minimization of influencing factors and errors occurring during the pre-analytical phase [1]. It has been shown that storage at room temperature (23 °C) and storage at 4 °C have an influence on numerous measured biomarkers, for example, for the activity of lactate dehydrogenase or the concentration of potassium and glucose [2]. The changes in the measured biomarkers result from the denaturation of enzymes and proteins, the lysis of cells (e.g., thrombocytes, erythrocytes, and leucocytes), and bacterial contamination. Furthermore, coagulation tests are subject to time-dependent changes in prothrombin time (PT) and the international normalized ratio (INR), as well as the activated partial thromboplastin time (aPTT), Factor V, fibrinogen, and D-dimers. Storage time prolonged for more than 8 h at room temperature between 18 °C and 24 °C leads to significant changes within those parameters [3]. A 24 h delay at room temperature or at 4–6 °C causes a negative change in the INR [4].

Time-dependent changes are of particular interest in outpatient care, as the time between blood collection and laboratory analysis is increased by the additional transport time. The exact time of blood taking is unknown to the laboratory. However, for individual interpretation of each biomarker, it is essential to know the delay in the examination of a specimen. Moreover, over 60% of errors occur during the pre-analytical phase [5]. This may influence further diagnostics and therapy and could result in higher therapy costs. The average cost per pre-analytical error is estimated to be approximately 200 USD [6].

Blood samples provide a fundamental basis for future research. The quality of prospective research depends on the quality of specimens in biobanks, in which large quantities of specimens (e.g., blood, DNA, other tissue samples) from a healthy population or from patients with specific diseases are stored over long periods. Kang et al. (2013) already showed that γ-glutamyl transferase (GGT) and lactate dehydrogenase (LDH) may be markers for the delay in specimen processing [7]. In practice, the usability of these biomarkers is limited for this purpose since the initial concentration differs from person to person. Furthermore, the results are dependent on the instrumentation and methodology used [7]. Because the interpretation of the data depends on the delay in testing a specimen, different delays in specimen testing in the same cohort could lead to misinterpretations.

Raman spectroscopy is a method of analyzing the complex composition of biological samples. Raman spectra primarily contain information about the vibrational excitation of specific molecular groups. In medical fields, Raman spectroscopy is becoming increasingly popular, and new methods relying on Raman spectroscopy are being developed. Forthcoming areas of application could include the diagnostics of many cancer entities [8], the staging of bronchial asthma as a consequence of changes in protein structures [9], malaria diagnostics [10], and the detection, identification, and circumstances of bacterial resistance to antibiotics [11,12]. Previous research suggests the advantage of Raman spectroscopy as a screening tool for a wide variety of biomarkers in serum. Precise predictions for the following markers could already be achieved for total bilirubin, conjugated bilirubin, unconjugated bilirubin, and total cholesterol, among others [13]. However, although much research is being conducted in this field, Raman spectroscopy is not yet incorporated into routine laboratory diagnostics.

## 2. Results

**Figure 1:**
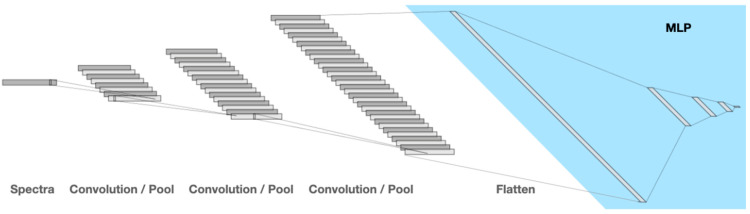
The architecture of the Convolutional Neural Network (CNN). Input are the spectra with 3438 frequencies, which are subjected to the following operations in three iterations: pattern detection (1D convolution), activation function (leaky ReLu), and data reduction (maximum pooling). The pattern detection window (kernel) size is 3 in each layer, and the convolution layers have 8, 16, and 32 descriptors (patterns), respectively. Finally, the output is flattened and input to the MLP (light blue) with 3 hidden layers of sizes 256, 128, 56 and as many outputs as classes to be predicted.

First, we identified the basic CNN settings suitable for our tests. We performed a series of optimizations using varying architectures of the CNN to determine one as small as possible yet able to reliably learn the delay of the samples from their spectra. We plotted the course of the accuracy of the training data. For this series, we used the entire data for the training. Runs that attained an accuracy of 100% within 100 k epochs in 20-fold cross-validations were considered successful. Table 1 presents the resulting architecture that was used in all subsequent runs, as further illustrated in Figure 1 (created using “https://alexlenail.me/NN-SVG/LeNet.html (accessed on 1 February 2023)”) [14].

Having identified a suitable basic architecture, we compared different ways of training CNNs with the collected data. Some tests utilized regularization, but mainly we relied on data modification. While neural networks (NN) do not provide insight into the underlying causalities, variations in the data used for training will still enable some understanding of the features of the dataset.

**Figure 2:**
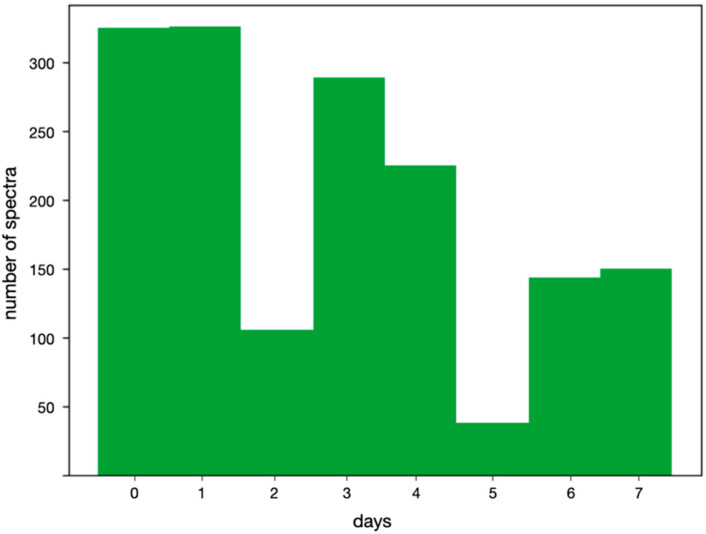
Size of datasets for each day.

The key modification to the data was the selection of days included in the training. Table 2 and the histogram in Figure 2 reveal that the number of spectra measured during the eight days represented far from an equal distribution and, thus, not a favorable starting point for machine learning. Days 2 and 5, especially, were strongly underrepresented.

Regarding the naming convention of the datasets, “all” includes the entire 1603 spectra without any modification. According to Figure 2, it is a rather unbalanced dataset. The other datasets are denoted first by the days explicitly contained in them. For example, “0, 1” contains spectra taken on day 0 and day 1, and “0, 1, 3 + 4” would additionally contain the spectra for days 3 and 4 merged into a single bin. Further, “0, rest” contains day 0 and all other remaining days merged into a single bin.

**Figure 3:**
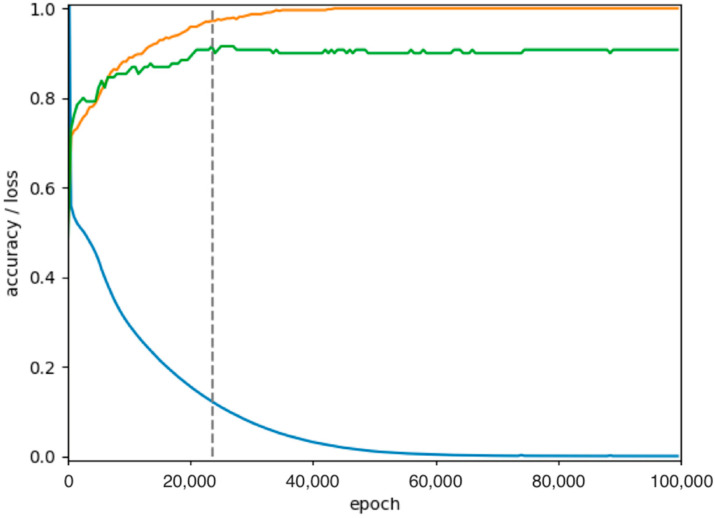
Evolution of loss function (blue, cross entropy), accuracy of training data (orange), and accuracy of the test data (green) over the iterations of the optimization (dataset “0, rest”). The dotted line indicates the maximum accuracy of the test data, after which any further decrease of loss only results in overfitting to the training data.

All datasets except for “all” are balanced by randomly erasing excess data to obtain a uniform distribution. This random filtering is part of each individual run and therefore differs for every cross-validation. If not otherwise specified, 20-fold cross-validation was performed per run. The boxplots show the statistical distribution of accuracies obtained, namely the minimum, maximum, median, and quartiles for the dataset under consideration. Accordingly, 20 values represented per boxplot was the default. Each individual value describes the final predictive power of the model for one run, which was chosen to be the maximum accuracy obtained for the independent test data. Figure 3 illustrates the time evolution of the loss function, driving the optimization and the accuracies of test and training data. The final predictive power would be the maximum of the green curve for the test data. Subsequent steps can be considered overfitting.

**Figure 4:**
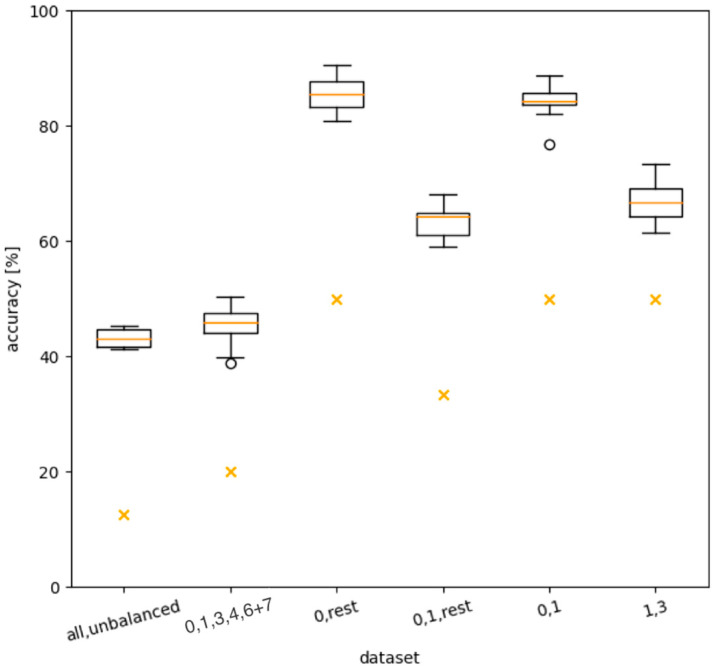
Statistical distribution of accuracies over 20-fold cross-validation for different datasets. The boxplots indicate median, quartiles, and limits. Additionally, the random value for the number of days included in the dataset is highlighted (as orange “×”). Numbers in labels on the x-axis indicate dataset (for example, “0, 1” contains spectra taken on day 0 and day 1; “0, rest” contains day 0 and all other remaining days merged into a single bin). The spheres represent data that extend beyond the whiskers.

Figure 4 shows the results for the first round of tests, all performed with the settings listed in Table 1. To illustrate the degree of learning of the algorithm, we added the accuracy that would be achieved if the predictions were purely random as a reference to the boxplots. The first plot on the left shows the *baseline* test using the “all” dataset, which achieves an accuracy of about 45%.

**Figure 5:**
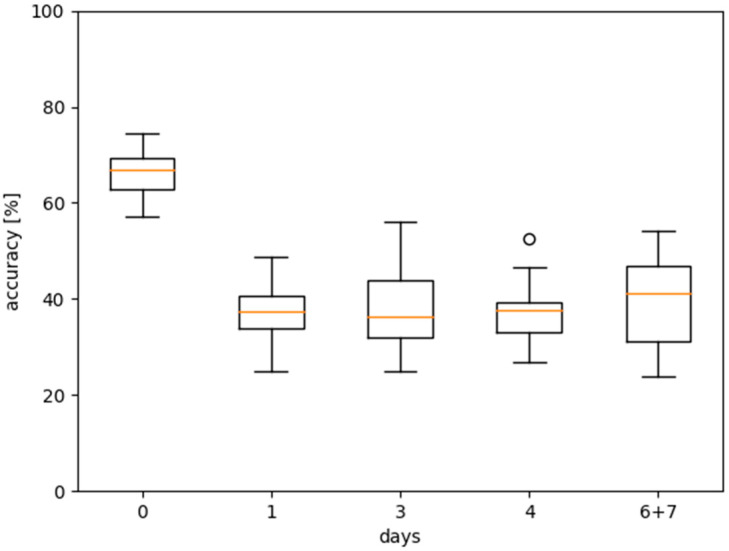
Accuracies over 20-fold cross-validation per day for dataset “0, 1, 3, 4, 6 + 7”.

As a second test, we omitted the underrepresented days 2 and 5 and additionally merged days 6 and 7. The dataset is denoted accordingly: “0, 1, 3, 4, 6 + 7”. As expected, this yielded slightly better results. Figure 5 shows the statistics of the per-day accuracies for this dataset, indicating that day 0 was consistently predicted more reliably than any other day, which all attained approximately the same level of accuracy. Therefore, distinguishing day 0 from the rest of the days should lead to the best results, as verified by the third plot in Figure 4. According to Figure 5, adding another day to the prediction should reduce the accuracy, which, again, the fourth plot verifies. We performed two additional, slightly more focused tests. The dataset “0, 1” attained a very similar accuracy to “0, rest”, while “1, 3” attained a similar accuracy to “0, 1, rest”. Since the samples were stored at room temperature from day 0 to day 1, the most significant biochemical changes in the sample occurred during this period.

**Figure 6:**
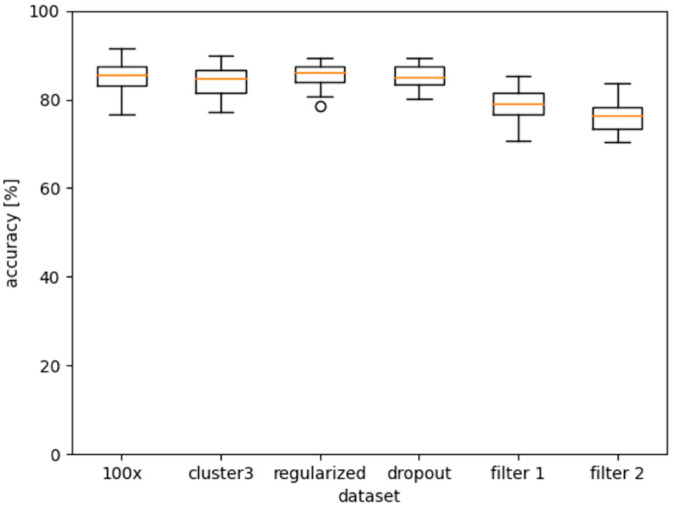
Further tests on the “0, rest’” dataset. The first plot from the left shows a 100-fold cross-validation. The second was performed on the largest cluster originating from spectral clustering. The third boxplot had a regularization factor of 1 × 10^−5^ defined. The fourth used a dropout probability of 0.2. For the fifth and sixth, we performed fluorescence filtering as defined in [13].

The second round of tests, shown in Figure 6, further investigated dataset “0, rest”, which clearly performed best. In the first plot, a 100-fold cross-validation was performed, confirming the results of the 20-fold cross-validation.

The difference in fluorescence in the raw data is highly dependent on liver function and can be partially explained by increasing bilirubin concentrations. The variation between the spectra of “healthy” versus terminally ill patients is dramatic and differs by more than an order of magnitude. Clustering of spectra, therefore, leads to the grouping of patients according to the stage of liver cirrhosis. We would have expected clustering to improve the learning of aging effects. We performed spectral clustering and trained on the largest cluster. Interestingly, no significant change was observed. Regularization and dropout also did not improve learning.

In Staritzbichler et al. (2021), we investigated a number of markers for which filtering the fluorescence background was required to obtain reliable predictions [13]. When we applied the same filter (“filter 2”) to the spectra of dataset “0, rest”, the accuracies dropped to some degree. “Filter 1” is a less flexible smoothing algorithm, while “filter 2” is the more rigorous filter. An increasing level of background filtering seems to directly lower the predictive power.

## 3. Discussion

Our patient cohort included patients with diagnosed liver cirrhosis. The cohort contained a variety of patients, ranging from a good state of health initially to patients in the final stage of liver disease, with strongly limited function. The loss in liver function results in a change in the blood composite, e.g., highly increased levels of fluorescing molecules such as bilirubin or reduction of albumin. Our observation that the filtering of fluorescence leads to a decrease in accuracy suggests that the aging process is reflected in some way in changes in fluorescence in this cohort. Simply transferring the delay prediction algorithm onto other patient cohorts (e.g., patients with renal or cardiac failure) could likely result in significantly less precise predictions. Therefore, further research with larger and cross-disease cohorts is needed. Spectra from cohorts associated with other diseases will provide further insight into the role of fluorescence in aging samples.

Although we cannot say at this point what exactly is driving the aging of the samples, a comparison of the accuracies for “0, rest” with “0, 1, rest”, “1–3”, and the per-day accuracies of “0, 1, 3, 4, 6 + 7” in Figure 5 revealed that changes were more distinct and therefore easier to learn between the first and the second day than between the second and the rest of the days.

All the samples measured were retention samples that had already been frozen and stored at −20 °C. No statement about the age of the specimen before the time of aliquotation of the retention sample can be made. Since, in our study, it was mainly the change within days that was analyzed as a marker, this should not affect the prediction significantly. To the best of our knowledge, the influence of freezing cycles on the Raman spectra has not been described so far.

Moreover, we used centrifuged serum blood samples for our investigations. We are not able to make a statement regarding cellular processes involved in the aging of a specimen. In this setup, the investigations were performed in samples without metabolic activity. Another interfering factor for the Raman spectra of the serum may be possible contamination with bacteria (e.g., during the process of blood collection or analysis in the laboratory). The rates of artificial bacterial contamination of blood cultures vary from 0.6% up to 6% [15]. Blood and serum are suitable growth mediums for bacteria, especially at room temperature. Refrigerator temperature slows the metabolism and growth of bacteria. The bacteria and their metabolites have specific spectra and can overlay the spectra of the serum sample with exponential growth. For this reason, the sample is stored at room temperature for only one day and at 7 °C from the second day. This could have caused the lower accuracy of delay discrimination within the last days.

We have shown that Raman spectroscopy could be a useful tool for predicting the delay in the analysis of blood specimens and acting as a method for quality assurance in the storage of samples in biobanks. However, further research is needed to optimize the measurement parameters and adjust the analysis of the spectra. For example, owing to the design of our instrument, it was necessary to place the samples on glass slides through which the spectra were recorded and which themselves created a background.

It was not the aim of this proof-of-concept study to derive a predictive model. Application of such a predictor in a clinical context would be highly premature. Although we had a fairly large cohort, this would require significantly more data across different cohorts and diseases. In this study, we were able to show that delay prediction was possible using Raman and fluorescence spectroscopy. This technique could one day be useful in improving the quality of biobanks and the research that emerges therefrom.

## 4. Materials and Methods

### 4.1. Samples and Spectra

In 2012, Leipzig University Hospital introduced a special quality assurance method for MELD diagnostics (Model of End-Stage Liver Disease). MELD is required for patients with liver cirrhosis eligible for a liver transplant at the time of initial diagnosis as well as in follow-up and progress examinations. As part of the quality assurance process, serum retention samples are taken from routine diagnostics and stored at −20 °C. We analyzed 330 of these serum samples using the Thermo Scientific DXR 3 SmartRaman Spectrometer (Thermo Scientific Waltham, MA, USA).

**Figure 7:**
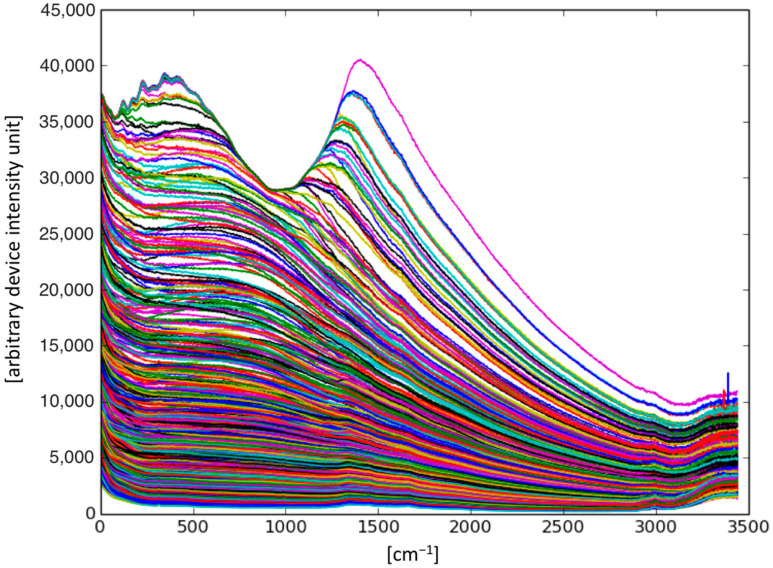
Overview of spectral data used in this study. The best learning performance was obtained with raw data, regardless of the massive background. The curves plot intensity (y-axis in arbitrary device-dependent units) against spatial frequency (x-axis in wavenumbers). Colors are random, for visual distinction only.

For the measurements, 50 µL of each sample was placed on a glass cover slip. The samples were measured using a 785 nm laser with a power of 150 mW. A two-cycle loop was used for the automatic measurement. The first loop included three sets with an exposure time of 10 s per set, and the second loop included two sets with an exposure time of 30 s for each set. Subsequently, each set was saved separately for individual analysis. We measured each sample on different days over the course of one week. Table 3 shows the storage conditions of the sample over the week. Table 4 describes the compilation of patients. Figure 7 shows the collected spectra.

### 4.2. Convolutional Neural Networks

The most prominent deep learning algorithms are the so-called neural networks. In their most basic architecture (often denoted as multilayer perceptron—MLP), they represent a concatenation of simple linear and nonlinear functions that can model highly complex relationships. The more modern and more complex neural architectures extend this concept, such as recurrent neural networks (RNNs), graph neural networks (GNNs), or convolutional neural networks (CNNs).

CNNs follow a hierarchical approach that is inspired by physiology; convolutional layers first recognize basic patterns, which are then used as input to a *classical* neural network. This makes them a powerful method for learning extremely complex data. Input to CNNs can be fixed-size data of any dimensionality, such as time series (1D grid of samples at regular intervals), images (2D grid of pixels), or even higher-dimensional data (3D voxels in MRT-scans, for example).

They are a first-rate algorithm for pattern recognition [16] and are often used to analyze images. Other applications include natural language processing [17] or financial time series analysis [18]. They are also a remarkably powerful tool for tasks related to the medical or biophysical field and have been used for zonal segmentation of prostates [19] or brain tumors [20] and in magnetic resonance imaging and organs in computed tomography images [21]. Newer architectures are also able to perform on both 2D and 3D data and can execute a similar task (e.g., segmentation) on different data (e.g., prostate and cardiac segmentation) [22]. The ability of convolutional networks to extract patterns from data is also useful when dealing with spectral data and has been used to detect radio signals [23] or visualize features and extract peaks from Raman spectra [24].

In the present case, the convolutional neural network was responsible for pattern recognition in the one-dimensional Raman spectra of serum samples, each consisting of 3438 data points. We opted for smaller kernel (pattern detection window) sizes and more convolutions (more filters are applied, and more complex shapes can be detected), following earlier advances in the field [25], favoring a deeper subsequent MLP. The MLP was responsible for classifying the delay of the sample with either two (“without delay”, “delayed”) or more classes (“day1”, “day2”, …).

The neural network architectures presented in this paper were built with Pytorch 1.7.1. Available data were split 80/20 into training and testing data with a k = 20 k-fold cross-validation. The training was conducted on an Nvidia GeForce RTX 2080, and both the convolutional layers and the MLP were optimized within the same training procedure. In the present training, we observed a high propensity of our network architectures towards overfitting, which we tried to combat by limiting network complexity and introducing regularization methods and dropout layers. In the end, careful modifications to the data, such as balancing, proved to be the most effective solution.

## Figures and Tables

**Table 1:** Pytorch CNN settings used for all subsequent tests.

Training/Test Data/Number of Spectra	80%/20%/1603
Number of convolution layers	3
Kernel sizes	3, 3, 3
Number of descriptors	8, 16, 32
Maximum pooling sizes	3, 3, 3
Number of MLP hidden layers	3
Sizes of hidden layers	256, 128, 64
Optimizer	AdamW
Activation function	Leaky ReLU
Learning rate	9 × 10^−7^
Loss function	Cross entropy

**Table 2:** Number of measured samples per day.

Day	0	1	2	3	4	5	6	7
Number of Measured Samples	330	330	106	294	230	38	144	155

**Table 3:** Handling and storage conditions of the serum samples over the period of a week.

day 0	sample was removed from freezer (−20 °C), thawed; 50 µL was measured and discarded	after measurement, stored at room temperature (22 °C) until next day
day 1	sample (50 µL) was measured and discarded	after measurement, stored in refrigerator at 7 °C until the next day
days 2–6	sample removed from refrigerator, 50 µL was measured and discarded	after measurement, stored in refrigerator at 7 °C until the next day
day 7	sample removed from refrigerator, 50 µL was measured and discarded	after measurement, disposal of sample

**Table 4:** Clinical data of patients.

	Female	Male	Total
Number of patients	137	193	330
Age (range) [years]	54.7 (31–70)	56.8 (21–77)	55.8 (21–77)
Bilirubin (range) [µmol/L]	70.9 (3.2–537.6)	87.6 (3–911.2)	80.7 (3–911.2)
Creatinine (range) [µmol/L]	96.8 (29–333)	131.4 (44–707)	117.0 (29–707)
INR (range)	1.5 (0.9–2.5)	1.45 (0.9–3.3)	1.47 (0.9–3.3)
MELD (range)	16.2 (6–39)	15.5 (6–40)	15 (6–40)

## Data Availability

Data used in this study can be found at: https://starbeachlab.org/data/spectra/raman/sample_age/data.zip.
